# Identification and Characterisation of the CircRNAs Involved in the Regulation of Leaf Colour in *Quercus mongolica*

**DOI:** 10.3390/biology13030183

**Published:** 2024-03-14

**Authors:** Yangchen Yuan, Xinbo Pang, Jiushuai Pang, Qian Wang, Miaomiao Zhou, Yan Lu, Chenyang Xu, Dazhuang Huang

**Affiliations:** 1 College of Landscape Architecture and Tourism, Hebei Agricultural University, Baoding 071000, China; 18730272192@163.com (Y.Y.); wangqian_hebau@163.com (Q.W.); 2 Hongyashan State-Owned Forest Farm, Baoding 074200, China; pxb15633717296@163.com (X.P.); p15733286929@163.com (J.P.); zmm15733239790@163.com (M.Z.); luyan8882024@163.com (Y.L.); 13930275112@163.com (C.X.)

**Keywords:** *Quercus mongolica*, leaf colour, anthocyanin, circRNA, hormones

## Abstract

**Simple Summary:**

In this paper, the mechanism of circular RNAs influence on leaf color of *Quercus mongolica* was proposed for the first time. It was determined that a total of 88 circRNAs were differentially expressed and five phytohormones were significantly altered. As a result, this article identified a total of 16 circRNAs that may be involved in regulating the color of *Quercus mongolica* leaves. CircRNAs may affect the color of *Quercus mongolica* leaves by interacting with auxin, cytokinin, gibberellin, ethylene, and abscisic acid. This provides a theoretical basis for the cultivation of new varieties of colorful foliage species.

**Abstract:**

Circular RNAs (circRNAs) are important regulatory molecules involved in various biological processes. However, the potential function of circRNAs in the turning red process of *Quercus mongolica* leaves is unclear. This study used RNA-seq data to identify 6228 circRNAs in leaf samples from four different developmental stages and showed that 88 circRNAs were differentially expressed. A correlation analysis was performed between anthocyanins and the circRNAs. A total of 16 circRNAs that may be involved in regulating the colour of Mongolian oak leaves were identified. CircRNAs may affect the colour of *Q. mongolica* leaves by regulating auxin, cytokinin, gibberellin, ethylene, and abscisic acid. This study revealed the potential role of circRNAs in the colour change of *Q. mongolica* leaves.

## 1. Introduction

*Quercus mongolica* (Fagaceae) is a deciduous broadleaf tree species that is mainly distributed in northern and northeastern China, Korea, the Russian Far East, the Korean Peninsula, and Japan [1]. *Q. mongolica* is a colourful plant with high ornamental value, and its leaves develop different colours during seasonal change, making it suitable as a roadside tree, courtyard tree, and green tree [2,3]. During production, coloured-leaf plants exhibit a variety of changes in leaf color during the color-changing period. Their specific manifestations include leaf color, the start time of leaf color change, and the duration of leaf color change. Therefore, in-depth research on the colour-changing mechanism of key coloured leaf tree species is crucial for selecting new plant varieties and garden tree species.

Circular RNA (circRNA) is created when linear RNA undergoes a “backsplice” between a downstream 3′ splice site and an upstream 5′ splice site. Spliced circRNAs range from <100 nt to >4000 nt [4] but are commonly a few hundred nucleotides [5]. CircRNAs are a class of endogenous non-coding RNA molecules widely present in eukaryotic cells. They do not have a 5′ terminal cap or a 3′ terminal poly (A) tail, and form closed circular structures through covalent circular RNA bonds [6]. Several studies have reported circRNAs in plants, such as Arabidopsis [7,8,9,10], soybean [11,12], rice [13], maize [14,15,16], tomato [17,18], barley [19], tea [20], cotton [21,22], and wheat [23,24,25].

As the main pigment affecting leaf discolouration, anthocyanins are produced by enzymes encoded by structural genes with phenylalanine. Among them, phenylalanine ammonia-lyase is the first enzyme in the synthetic pathway, and changes in its activity play a crucial role in the synthesis of phenylalanine compounds [26]. Dihydroflavonol-4 reductase catalyses the production of colourless anthocyanins from dihydroflavonols, which is a key regulatory point determining the transition of anthocyanins from colourless to coloured [27]. Anthocyanin synthase is located at the end of the anthocyanin glycoside synthesis pathway and is the key enzyme that catalyses the conversion of colourless anthocyanins to coloured anthocyanins [28]. Flavonoid 3-O-glucosyl transferase converts unstable anthocyanins to stable anthocyanosides in a glycosylation reaction [29]. All of these enzymes play crucial roles in anthocyanin synthesis, affecting the colouration of the leaves of coloured plants. The colouration mechanism of coloured leaf species is complex, and factors such as pigment content, pigment type, and pigment distribution within the leaf cause changes in leaf colouration [30]. Transcription factors play an important role in the synthesis of pigments [31]. Anthocyanins change exist at any stage of oak leaves’ growth and development. However, the accumulation of anthocyanins in oak leaves is visually detected at later stages of its development.

A study of metabolites during senescence (yellowing) of *Q. mongolica* leaves found that differential metabolites during the senescence stage were mainly enriched in amino acid metabolism, lipid metabolism, and the biosynthesis of secondary metabolites [32]. However, current research on the colour change mechanism of red leaf color change in *Q. mongolica* leaves is insufficient, and the relationship between circRNAs and the change in *Q. mongolica* leaf colour has not been reported. Therefore, in this study, we chose four different developmental stages of *Q. mongolica* to analyze the changing leaf pigment patterns, the leaf color parameters, and hormone contents. Differentially-expressed circRNAs (DECs) were identified and screened, and their enrichment was analysed. DECs were identified and further validated using real-time polymerase chain reaction (PCR). This is the first report to identify differentially expressed circRNAs in *Q. mongolica* leaves at different times (four developmental stages). The results will help to investigate further the potential role of circRNAs in regulating leaf colour in *Q. mongolica*.

## 2. Materials and Methods

### 2.1. Plant Materials

**Figure 1:**
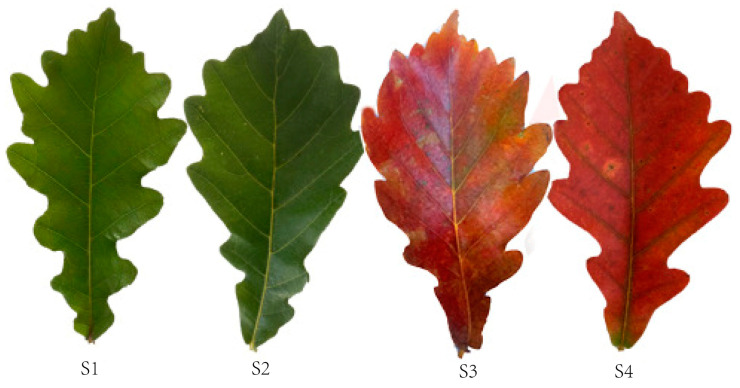
*Q. mongolica* phenotypes during the four developmental stages: young leaf stage (S1), green leaf stage (S2), colour change stage (S3), and red leaf stage (S4).

The study site was located at Caijiayu, Yixian County, Baoding City, China (39°32′6′′ N, 113°52′10′′ E, 1400 m above sea level). We screened nine *Q. mongolica* trees with good and stable growth conditions, aged 15–20 years, in a natural Mongolian oak forest. Leaf samples were collected during four growth periods: 10 May (S1), 4 August (S2), 9 October (S3), and 18 October (S4) in 2022 (Figure 1). Due to the fact that the trees we are studying grow in open field environments, there is no significant difference in the process of leaf color change in all directions. However, in order to ensure the scientific validity of the experiment, we still collected functional leaves from the central part of the current year branches from the east, west, south, and north parts of the tree canopy for research. The collected leaf samples were immediately frozen in liquid nitrogen and stored at a temperature of −80 °C. The leaf samples were collected from all nine trees at each stage of growth. Leaves from nine trees at each stage were mixed with three biological replicates for a total of 12 samples. We divided leaf growth and development into four stages: the young leaf stage (S1), the green leaf stage (S2), the color change stage (S3), and the red leaf stage (S4).

### 2.2. Measurement of Leaf Colour Parameters

The leaf colour parameters were analysed using a colourimeter (CR-400; Konica Minolta, Tokyo, Japan). Leaf colour was measured during four different developmental stages. 30 leaves were randomly collected from four directions of the tree canopy for measurement. Ten leaves with similar growth were selected. The colour characteristics of 10 leaves were measured at the tip and centre of the leaf as well as at the base of the petiole to obtain an average value. These measurements were repeated three times, and the L*, a* and b* values were recorded. L* is the color brightness, from 0 to 100, that is, from black to white. The hue a* represents the green-red axis, ranging from −120 to 120, and the color ranges from green to red from the negative axis to the positive axis. The hue b* represents the blue-yellow axis, ranging from −120 to 120. Negative values indicate the degree of blue, while positive values indicate the degree of yellow [33].

### 2.3. Determination of the Physiological Indicators

Chlorophyll and carotenoid concentrations were determined by direct ethanol extraction [34]. First, the fresh leaves were washed with distilled water and the veins of the leaves were removed. The leaves were weighed to 0.1 g and cut into 1 mm wide strips. The cut leaves were placed in a test tube containing 10 mL of 95% ethanol. Then, the test tube was sealed with plastic wrap and stored in the dark for 12–24 h until the leaf strips turned completely white. At last, the solution was aspirated into a cuvette. Chlorophyll and carotenoid concentrations were calculated by measuring the optical density values at 665, 649 and 470 nm with a spectrophotometer, with 95% ethanol as the blank control.

The anthocyanin content was determined using the method of Li et al. [35]. Fresh leaves were cut and weighed to the nearest 0.1 g in a triangular flask containing 10 mL of 1 mol L^−1^ HCl. The mixture was placed in an oven at 32 °C for 8 h, and centrifuged. The supernatant was collected to determine the optical density at 530 nm and to calculate anthocyanin content.

Enzyme-linked immunosorbent assay kits (Quanzhou Ruixin Biotechnology Co., Ltd., Quanzhou, China) were used to measure leaf indole acetic acid (IAA), gibberellin (GA3), cytokinin (CTK), ethylene (ET) and abscisic acid (ABA) contents, according to the manufacturer’s instructions.

### 2.4. RNA Extraction, Library Construction and Sequencing

First, the total RNA was extracted using a Trizol kit (Invitrogen, Carlsbad, CA, USA) according to the manufacturer’s method. The quality of the extracted RNA was then assessed on an Agilent 2100 Bioanalyzer (Agilent Technologies, Palo Alto, CA, USA). RNase-free agarose gel electrophoresis was used for examination. The extracted total RNA was followed by the removal of the rRNA and retention of ncRNA and mRNA. Next, the retained mRNA and ncRNA were fragmented into short fragments using a fragmentation buffer, and reverse transcribed into cDNA using random primers. Second-strand cDNA was synthesised with DNA polymerase I, RNase H, dNTP (dUTP instead of dTTP) and buffer. The cDNA fragments were purified with the QiaQuick PCR extraction kit (Qiagen, Venlo, The Netherlands), end-repaired, poly(a) tails were added, and ligated to the Illumina Sequencing Adapter. The second-strand cDNA was then digested with uracil-N-glycosylase. Finally, the digested products were screened for size by agarose gel electrophoresis, amplified by PCR, and sequenced using the Illumina HiSeqTM 4000 from Gene Denovo Biotechnology (Guangzhou, China).

### 2.5. Identification of CircRNA

The 20 mers from both ends of the unmapped reads were extracted and aligned to the reference genome to identify the unique anchor positions within a splice site. Anchor reads that aligned in the reversed orientation (head-to-tail) indicated splicing of circRNA and were subjected to CIRIquant to identify the circRNAs [36]. A candidate circRNA was called if it was supported by at least two unique back-spliced reads in at least in one sample [37].

### 2.6. Analysis of Differentially Expressed CircRNAs

To identify the differentially expressed circRNAs across samples or groups, the edgeR package (4.0.2) [38] was used. We identified circRNAs with |log2FC| > 1 and a *p*-value < 0.05 in a comparison between samples or groups as significantly differentially expressed circRNAs.

### 2.7. Real-Time PCR Analysis

We used DNase I (NEB, Beijing, China) to remove DNA contamination from the RNA samples used to validate circRNA. Ribosomal RNA was removed following the steps of the Epicentre Ribo-Zero Gold Kit (Illumina, San Diego, CA, USA) and linear RNA was removed by incubation with 3 units μg^−1^ of RNase R (Epicentre) at 37 °C for 15 min. Two sets of primers were designed for each circRNA using Primer3. An outward-facing set was expected to amplify only the circRNAs across the back-spliced junction (divergent primers), and an opposite-directed set was expected to amplify the linear mRNA forms (convergent primers). Experimental expression levels of circRNAs were quantified on a StepOnePlus real-time PCR System (Thermo Fisher Scientific, Wilmington, DE, USA) using the MagicSYBR Mixture (Hangzhou, China), in which the cDNA was synthesised using random 6-mers. The relative expression ratio (ΔCt) of each circRNA was calculated using the 2^−ΔCt^ method. As in previous qRT-PCR experiments [39,40], GAPDH was used as an internal standard control and all reactions were performed in triplicate. Convergent and divergent primers for qRT-PCR were designed using Primer 3 web (http://primer3.ut.ee/) to amplify linear mRNA and circRNAs forms.

### 2.8. Enrichment Analysis of Parental DEC Genes

To evaluate the potential functions of the circRNAs, their parental mRNAs were used for BLAST searches and functionally categorised according to GO and KEGG pathway databases.

### 2.9. Statistical Analysis

Statistical analysis of the leaf physiological data was performed with IBM SPSS Statistics 23 software (IBM Corp., Armonk, NY, USA) using the analysis of variance and Duncan’s test to determine significant differences between samples. A *p*-value < 0.05 was considered significant. Bar graphs, Venn diagrams, and correlation heatmaps were prepared using the omicshare online tool (https://www.omicshare.com/tools/Home/Soft/getSoft, accessed on 20 January 2024).

## 3. Results

### 3.1. Changes in the Leaf Colour Parameters

**Figure 2:**
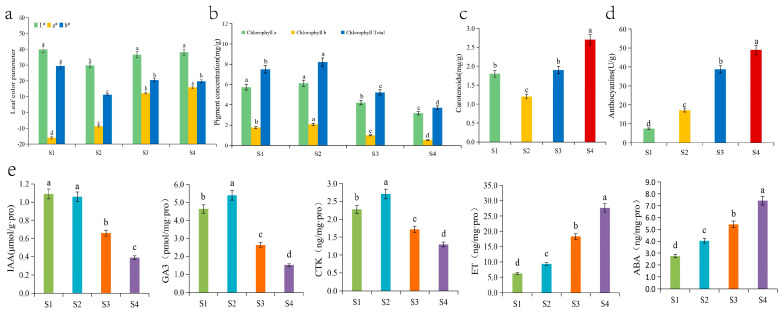
Changes in the (**a**) leaf colour parameters, (**b**) chlorophyll contents, (**c**) carotenoid content, (**d**) anthocyanin content, and (**e**) five hormone contents of *Q. mongolica*. Different lowercase letters indicate significant differences between the groups (*p* < 0.05).

The leaf colour parameters changed during the development of *Q. mongolica* leaves and the leaf colour parameter L* decreased significantly, followed by a significant increase and a levelling off during the leaf development. No significant difference between the leaf colour parameters was observed during the S1, S3 and S4 periods, but they were significantly higher than those during the S2 period, indicating that leaf brightness was higher in young leaves, and during the colour transition and senescence periods, than during the green leaf period. The leaf colour parameter a* increased significantly during leaf development, indicating a change in leaf colour from green to red. Leaf colour parameter b* decreased significantly and then increased significantly during leaf development, with S1 as the highest point, S2 as the lowest point, and S3 and S4 with a stable trend and no significant changes. The young leaf stage, the colour change stage, and the senescence stage were significantly higher than those in the green leaf stage. It is hypothesized that it may be due to the low chlorophyll content of leaves in the S1 stage and the large accumulation of secondary metabolites such as flavonoids, which resulted in a higher degree of yellow coloration of leaves in the S1 stage than that in the green leaf stage (S2) and red leaf stage (S3, S4). The values for S3 stage and S4 stage were higher than those for S2 stage, indicating that leaf yellowness was greater during the color change stage and red leaf stage than the green leaf stage (Figure 2a).

### 3.2. Changes in Leaf Pigment Content

The chlorophyll content of *Q. mongolica* leaves increased and then decreased over time. Chlorophyll a content increased slightly followed by a significant decrease during the four stages. Chlorophyll b content increased significantly followed by a significant decrease, reaching a peak at the S2 stage. The total chlorophyll first significantly increased, then significantly decreased, reaching its peak in the S2 phase and reaching its trough in the S4 phase. The content of total chlorophyll in the first three stages was 2.03, 2.22, and 1.41 times higher than that in the S4 stage, respectively. The content of carotenoids significantly decreased over time, followed by a significant increase, reaching a valley in the S2 phase and a peak in the S4 phase. The content of carotenoids in the first three stages was 0.67, 0.44, and 0.70 times higher than that in the S4 stage, respectively. The ratios of carotenoids to total chlorophyll at the four stages were 0.24, 0.15, 0.37 and 0.73, respectively (Figure 2b). The anthocyanin content in *Q. mongolica* leaves increased significantly over time, particularly at the S4 stage, which was more than 6.5 times higher than that at the S1 stage (Figure 2c). Overall, the anthocyanin content in the leaves increased significantly during development. Chlorophyll content increased and then decreased, and carotenoid content decreased and then increased significantly.

### 3.3. Hormonal Changes

In this study, we measured the content of five hormones in *Q. mongolica* leaves at different periods: the IAA content was similar in the S1 and S2 stages, and then showed a significant decreasing trend. It peaked in the S2 stage and reached a trough in the S4 stage, and the content in the S2 was 2.7 times higher than that in the S4. GA3 content showed a trend of significant increase followed by significant decrease, reaching the maximum and minimum values in the S2 and S4 periods, respectively. The GA3 content in the first three periods was 3.05, 3.55, and 1.73 times higher than that in the S4 period, respectively. The trend of CTK content was similar to that of GA3, which also showed a significant increase and then a significant decrease, reaching the maximum and minimum in the S2 and S4 stages, respectively. The CTK contents in the first three periods were 1.76, 2.1, and 1.33 times higher than those in the S4 period, respectively. Among the five phytohormones, ethylene had the highest relative content and showed a significant increase with leaf growth and development. Its maximum value was as high as 27.67 ng/mg.pro in the S4 period, which was 4.43, 2.97, and 1.51 times higher than the previous three periods, respectively. The trend of ABA content was similar to that of ethylene, showing a significant increase. However, the content was much lower than that of ethylene in the same period (Figure 2d).

### 3.4. CircRNA Identification

**Figure 3:**
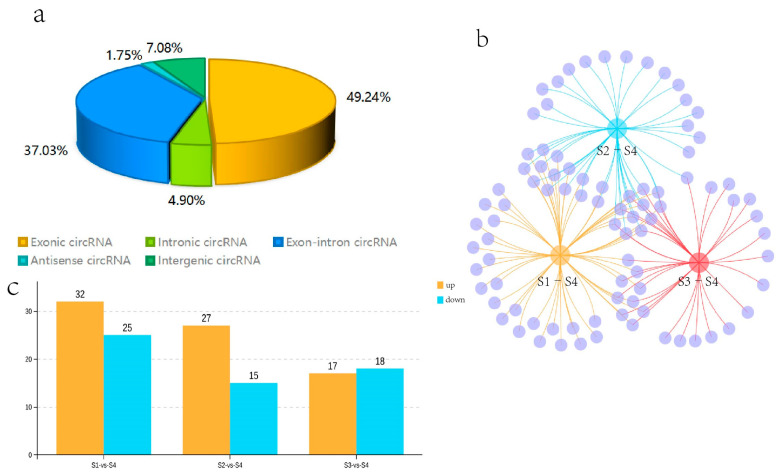
Statistical analysis of the identified circRNAs and the differentially expressed circRNAs (DECs) in S1, S2, S3, and S4. (**a**) The proportions of the various types of circular RNAs. (**b**) Venn diagram analysis of the DECs in S1–S4, S2–S4, and S3–S4. (**c**) The number of upregulated and downregulated DECs in each comparison.

Twelve cDNA libraries from the *Q. mongolica* S1, S2, S3, and S4 stages were constructed and sequenced to determine the circRNAs involved in the regulation of leaf colour. In all samples, 70.74–84.16% of the reads were successfully mapped to the *Q. mongolica* genome. HISAT2 was employed to detect the head-to-tail splicing (back-spliced) of the remaining 15–24% of the unmapped RNA-seq reads. A total of 6228 circRNAs were identified. Among them, 49.24% of the circRNAs were exon circRNAs. The ratio of antisense circRNA was the lowest, at 1.75%, while intron, intergenic and exon-intron circRNAs were 4.90%, 7.08%, and 37.03%, respectively (Figure 3a).

### 3.5. Identification of Differentially Expressed CircRNAs

We compared the expression of circRNAs in S1, S2, S3, and S4, and obtained the DECs. Fifty-seven circRNAs were identified as DECs in the S1–S4 comparison (*p*-value < 0.05 and |log2FC| > 1). 32 circRNAs were upregulated and 25 were downregulated in S1 compared with S4 among them. We identified 42 DECs in the one-to-one comparison between S2 and S4. Among them, 27 circRNAs were upregulated and 15 were downregulated in S2 compared with S4. Thirty-five DECs were specifically found in the S3–S4 comparison. Among them, 17 circRNAs were upregulated and 18 circRNAs were downregulated in S3 compared with S4. In addition, the DECs that were common or unique to the three groups, such as S1–S4, S2–S4, and S3–S4, were visualised with a Venn diagram. Eleven DECs were common to the three groups; 14, 1, and 9 DECs were common to the two groups, and 23, 16, and 14 DECs were unique to the three groups, respectively (Figure 3b,c). Among the identified circRNAs, 11 DECs were present in all three comparative groups. Due to the significantly redder leaf colour during S4 than S1–S3, the 11 coexisting DECs in these three comparisons were likely to be involved in the regulation of leaf colour in *Q. mongolica*.

### 3.6. Enrichment Analysis of the DEC Parental Genes

A total of 88 DECs were used to extract their parental genes for functional analysis based on the alignment of circRNAs with reference genomic locations. Of these, 77 DECs were predicted to have 69 parental genes and were annotated, while no parental coding genes were found in the remaining 11 DECs. The parental genes of the circRNAs were involved in biological processes, such as metabolic processes (GO:0008152), signalling (GO:0023052), biological regulation (GO:0065007), and regulation of biological processes (GO:0050789) as well as in cellular components, such as protein-containing complex (GO:0032991) and cellular anatomical entity (GO:0110165), and molecular functions, such as transporter activity (GO:0005215), catalytic activity (GO:0003824), ATP-dependent activity (GO:0140657), and binding (GO:0005488). The KEGG enrichment analysis revealed that the differentially expressed genes were centrally enriched in sesquiterpene and triterpene biosynthesis (Ko00909), secondary metabolite biosynthesis (Ko01110), monoterpene biosynthesis (Ko00902), sulphur metabolism (Ko00920), phytohormone signalling (Ko04075), metabolic pathways (Ko01100), amino acid biosynthesis (Ko01230), and glycolytic gluconeogenesis (Ko00010) (Supplementary Figure S1).

### 3.7. Correlation Analysis

**Figure 4:**
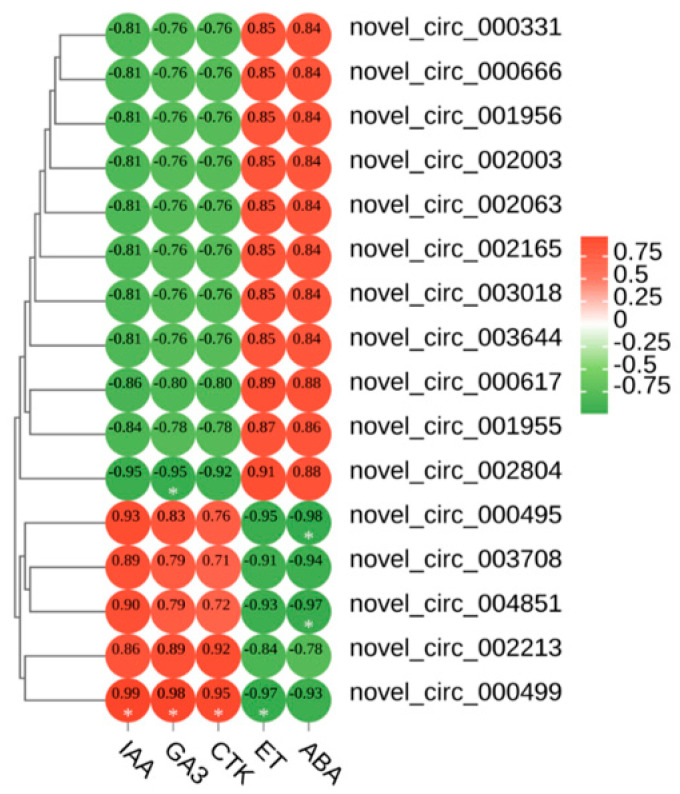
Heatmap of the correlations between the 5 hormones and 16 DECs. * Indicates significant correlation (*p* < 0.05).

We correlated the leaf colour parameters, pigment content, and five phytohormones with the circRNAs (Supplementary Figure S2). The results showed that novel_ circ_ 004851, novel_ circ_ 003708, novel_ circ_ 000499, and novel circ_ 000495 were highly significantly negatively correlated with anthocyanins. Novel_ circ_ 002804 was highly significantly positively correlated with anthocyanins. We speculated that these five DECS are involved in the regulation of leaf colour in *Q. mongolica*. To investigate the effects of the five hormones on leaf colour, we conducted a correlation analysis between IAA, GA3, CTK, ET, ABA and 16 DECs. As a result, ABA and novel_ circ_ 004851 and novel_ circ_ 000495 were highly significantly negatively correlated. ET and novel_ circ_ 000499 were highly significantly negatively correlated. IAA, GA3 and CTK were highly significantly positively correlated with novel_ circ_ 000499. GA3 and novel_ circ_ 002804 were highly significantly negatively correlated (Figure 4).

### 3.8. Real-Time PCR Verification of the DECs

**Figure 5:**
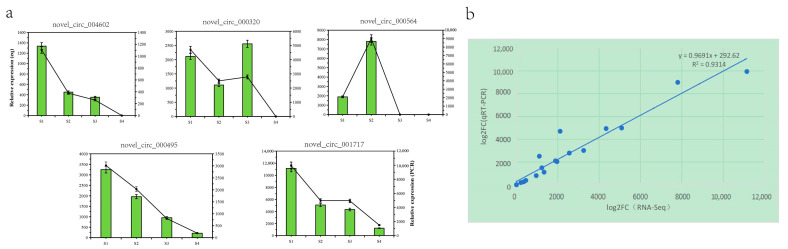
Validation of *Q. mongolica* (**a**) RNA-seq data and qRT-PCR results through (**b**) comparisons of the log2 gene expression ratios between the RNA-seq and qRT-PCR results, respectively.

To confirm further the reliability of the RNA seq data, we randomly selected 5 genes from the 88 differential genes for RNA seq data validation. The results indicated a similar expression trend between the RNA-seq data and the qRT-PCR results (Figure 5a). Linear regression analysis revealed a significant positive correlation between the qRT-PCR and RNA-seq data (Figure 5b), indicating the accuracy and reliability of the RNA-seq data.

## 4. Discussion

CircRNAs are widely present in plants and play important roles in regulating growth, development, and stress tolerance [7,8,9,10,11,12,13,14,15,16,17,18,19,20,21,22,23,24,25]. However, whether it is involved in the regulation of leaf color is not clear. In this study, we investigated this question in terms of hormone content and cyclic rna.

The leaf color parameters of plants can quantitatively respond to the color of leaves, making it more intuitive and convenient to observe the leaf color changes. Studies on different seed sources of *Euonymus europaea* showed that the a* value was highly significantly negatively correlated with chlorophyll content and significantly positively correlated with anthocyanin glycoside content [41]. In the present study, a* showed highly significant positive correlation with anthocyanins, which is consistent with the above study. In a study of three different varieties of *E. europaea*, it was shown that there was no significant relationship between L* and chlorophyll, carotenoid, and anthocyanin content of the three varieties, respectively. The same was true for b*. However, a* was highly significantly positively correlated with anthocyanin content in three varieties, and a* was also highly significantly negatively correlated with carotenoids in one of the varieties named ‘Pumilis’. In a study of *Pistacia chinensis* Bunge, it was found that b* of *P. chinensis* from the Beijing provenance was not significantly related to chlorophyll, carotenoids, and anthocyanin content [42]. However, b* and anthocyanin content of the other three provenances of *P. chinensis* showed highly significant negative correlations. In the present study, L* was not significantly related to chlorophyll, carotenoid, and anthocyanin content, which is consistent with the study of *E. europaea*. b* was also found to be so. We hypothesize that the correlation between leaf color parameters and pigment content is not only related to species, variety, and provenances, but also possibly to climatic factors such as moisture, temperature, and light, which needs to be further verified.

The anthocyanin content continued to increase as the leaves developed, particularly during S3 and S4, which had significantly higher levels, indicating that anthocyanins accumulated rapidly in the fall when the leaves changed to red. Chlorophyll content increased and then decreased, indicating that chlorophyll continued to accumulate during leaf development, and the content was highest in mature leaves. Chlorophyll continued to degrade in the fall, and anthocyanins accumulated rapidly, which caused the leaves to appear red. Carotenoid content decreased and then increased. The carotenoids increased significantly, particularly during the S4 period, indicating that carotenoids accumulated significantly during the late stage of leaf development. This is consistent with the study of *P. chinensis* from Hebei provenance [42].

In addition to environmental factors, such as temperature and light, that affect anthocyanoside biosynthesis, hormones such as auxin (IAA), cytokinin (CTK), gibberellin (GA), ethylene (ET), and abscisic acid (ABA) affect anthocyanoside synthesis. The results of the study showed that growth hormone possessed a duality on anthocyanin synthesis, i.e., low concentration promotes and high concentration inhibits [43,44,45]. Studies have shown that in the callus tissue of Oxalis linearis [43], NAA repressed anthocyanin production with an increase in NAA from 8–32 μM. Anthocyanin synthesis was promoted by an increase in 2,4-D from 0.5 to 2 μM and decreased thereafter up to a concentration 32 μM. In this study, the concentrations of IAA in the S1, S2, and S3 stages were significantly higher than those in the S4 stage, and were 2.79, 2.72, and 1.69 times higher than those in the S4 stage, respectively. We hypothesized that high concentrations of IAA inhibited anthocyanin accumulation in *Q. mongolica* leaves. This inhibitory effect was weakened with decreasing IAA concentrations. Even when the IAA was as low as a critical value, it may have promoted anthocyanin accumulation. However, this requires further in-depth study.

Das et al. suggested that cytokinins increase the expression of structural genes, which in turn promotes anthocyanin biosynthesis [46]. However, some studies have also shown that cytokinins negatively regulate the synthesis of anthocyanins. For example, BA treatment significantly inhibits sucrose-induced anthocyanin synthesis in (non-chlorophyllou) corn leaves [47]. Similarly, 6-BA and CPPU treatments also inhibited anthocyanin accumulation in litchi pericarp, and gene expression analysis revealed that CPPU suppressed the expression of UFGT and its key regulator gene, LcMYB1 [48,49,50]. In this study, cytokinin concentration decreased significantly during the transition from green to red leaves, while anthocyanin content continued to increase. It is hypothesized that cytokinin has an inhibitory effect on leaf anthocyanin content and that this inhibitory effect diminishes with decreasing cytokinin concentration.

Gibberellins are widely used in agricultural production and also have an important effect on the biosynthesis of plant anthocyanins. Some studies have shown that gibberellin can promote anthocyanin accumulation. Hyacinthus accumulates anthocyanins only in the L2 epidermal cells of the perianth, but when flower buds were cultured in a medium containing GA3, both L1 and L2 epidermal cells of the perianth accumulated anthocyanins [51]. Exogenous GA3 treatment induces the expression of CHS gene in the corolla of petunia and promotes the accumulation of anthocyanins [52]. However, there are also studies indicating that GA inhibits the biosynthesis of anthocyanins. Ilan and Dougall (1992) treated carrot suspension culture cells with GA3 and significantly inhibited anthocyanin accumulation [53]. In the present study, GA3 concentration decreased significantly during the transition from green to red leaves, while anthocyanin content continued to increase. It was hypothesized that GA3 had an inhibitory effect on the anthocyanin content of leaves, and this inhibitory effect gradually weakened with the decreasing concentration of GA3.

Ethylene also has both positive and negative regulatory effects on anthocyanins. Studies have shown that ET inhibits the expression of positive regulatory genes (TT8, GL3, and PAP1) for anthocyanin synthesis in Arabidopsis, induces the expression of negative regulatory gene MYBL2, and thus inhibits sucrose induced anthocyanin synthesis [54]. El-Kereamy et al. treated grapefruits with 2-chloroethylphosphonic acid, which induced the upregulation of anthocyanin synthesis structural genes CHS, F3H, LDOX, and UFGT, and increased the anthocyanin content [55]. In this study, the trend of ethylene concentration was almost the same as that of leaf anthocyanin content with leaf growth and development, and the concentration of ethylene was much higher than that of other hormones in the same period. This shows that ethylene is regulating leaf anthocyanin accumulation, and this role may be crucial.

Abscisic acid has a positive regulatory effect on the synthesis of anthocyanins. It has been shown that exogenous ABA treatment induces the expression of structural and regulatory genes for anthocyanin synthesis and promotes anthocyanin accumulation in grape pericarp and cell suspension lines [56]. By studying lychee browning, Qu et al. found that the treatment of litchi with ABA up-regulated the activities of PAL, CHS, and UFGT, key enzymes involved in the anthocyanin synthesis pathway, and promoted the accumulation of anthocyanins [57].

At present, the mechanism of the phytohormone regulation of anthocyanins is not entirely clear. One study, Ni et al. on red pear fruit, showed that ethylene reduces anthocyanin synthesis by inhibiting the expression of structural genes encoding enzymes through repressing the expression of transcription factors PpMYB10 and PpMYB114 [58]. It has been shown that hormones can up-regulate the activities of key enzymes involved in the anthocyanin synthesis pathway and promote anthocyanin accumulation. Exogenous GA3 treatment induced the expression of the corolla CHS gene in Petunia (*Petunia kybrida*) and promoted anthocyanin accumulation. In the Arabidopsis gai mutant, it was shown that GA regulates anthocyanin biosynthesis through the DELLA protein [52]. In summary, phytohormones can regulate anthocyanins through transcription factors, structural genes, enzymes, and encoded proteins. In the present study, hormones may influence leaf anthocyanin biosynthesis via circRNA. However, it is not entirely clear that the detected increase in various phytohormones is a sensitive half-increase. It is also not clear whether phytohormones affect anthocyanin synthesis through the pathways described above, or by increasing antioxidant capacity or other pathways. Phytohormones may regulate anthocyanin synthesis in a variety of ways, and their mechanism of action on anthocyanin synthesis in Mongolian oak leaves is still unclear, which needs to be further investigated.

In this study, 6228 circRNAs were identified in the samples, and >49% belonged to the eciRNA class. ciRNAs were only 4.9% of the total number of circRNAs. The proportion of ciRNAs is similar to the results previously reported in wheat (<6.5%), *A. thaliana* (3.8%), and tomatoes (3.6%) [24,59,60]. Many studies have shown that circRNAs are involved in the regulation of gene expression by affecting their linear copies and regulating the transcription of their parental genes [61]. Huang et al. investigated the expression of circRNAs associated with the senescence process in rice rapeseed leaves and reported that 17 senescence-related circRNAs were predicted to have parental genes by weighted gene co-expression network analysis (WGCNA), of which regulation of their parental genes by three circRNAs was verified by qRT-PCR [62]. The expression of *AfNAC083* in *Acer fabri* is positively correlated with anthocyanin content [63]. Our analysis of the DECs and their parental genes showed that the parental gene of novel_circ_003018 is gene-Qm012601 (NAC083), which is an important transcription factor regulating leaf colour in *Q. mongolica*. We hypothesised that novel_circ_003018 is involved in anthocyanin biosynthesis through the regulation of the parental gene. The parental gene of Novel_circ_000499 was gene-Qm017453 (NAP1), and overexpression of NAP1 promotes leaf senescence in Dianthus caryophyllus [57] and Panicum virgatum [58]. The accumulation of anthocyanins in the leaves of *Q. mongolica* is not distinguishable from senescence, and it is hypothesised that NAP1 affects the accumulation of anthocyanins in *Q. mongolica* leaves, which requires further investigation.

Transcripts that contain multiple miRNA binding sites and inhibit miRNA activity are known as miRNA sponges. The results of animal studies indicate that the most significant function of circRNAs is to act as miRNA sponges or participate in miRNA-related pathways to regulate gene expression [64,65,66]. Although most circRNAs in plants do not have a large number of miRNA binding sites, they also act as miRNA sponges and regulate gene expression by binding miRNAs. Lu et al. showed that 235 circRNAs had miRNA binding sites in rice, among which 31 circRNAs had two or more putative miRNA binding sites [13]. Zeng et al. [67] studied the early flowering mutant of *Citrus aurantium* and reported 176 differentially expressed circRNAs compared with the wild-type, of which 29 circRNAs could be potential targets of 16 miRNAs. Capelari et al. identified five circRNAs in *A. thaliana* flowers with possible miRNA sponge functions, and the circRNAs were identified as potential targets by the wild-type [68]. In this study, we attempted to sequence miRNAs in *Q. mongolica* leaves to predict their binding sites with circRNAs, but this attempt was unsuccessful. We hypothesised that this result might be due to plant specificity, and we will continue to address the difficulties and carry out further studies.

## 5. Conclusions

Our study showed that the expression levels of some circRNAs differed significantly in *Q. mongolica* leaves at different developmental stages. Anthocyanins, growth hormones, CTK, GA, ABA, ET, and DECs were correlated. We successfully identified 16 DECs involved in the regulation of leaf colour in *Q. mongolica* and verified the circRNAs using qRT-PCR. CircRNAs regulated anthocyanin synthesis in *Q. mongolica* leaves and affected leaf colouration by regulating hormone levels. These results provide new clues for investigating the role of circRNAs in the regulation of colour in *Q. mongolica* leaves.

## Data Availability

This project was registered under the BioProject accession number PRJNA1076348, and the SAMN39939286 and SAMN39939297 Biosample accession numbers.

## Supplementary Materials

The following supporting information can be downloaded at: https://www.mdpi.com/article/10.3390/biology13030183/s1, Figure S1: (a) Bar chart for Gene Ontology enrichment analysis of three comparison groups. (b) Significance bubble plots for Kyoto Encyclopedia of Genes and Genomes (KEGG) analysis of three comparison groups; Figure S2: Heat map of leaf color parameters, pigment content and five phytohormones in relation to circRNAs.
