# Supplementary material for: Identification and Characterisation of the CircRNAs Involved in the Regulation of Leaf Colour in Quercus mongolica
Source: Biology (Basel). 2024 Mar 14;13(3):183. doi: 10.3390/biology13030183 (PMC10968399; doi:10.3390/biology13030183)
Supplement: Supplementary file 1 [file biology-13-00183-s001.zip › biology-2895164-supplementary.pdf]

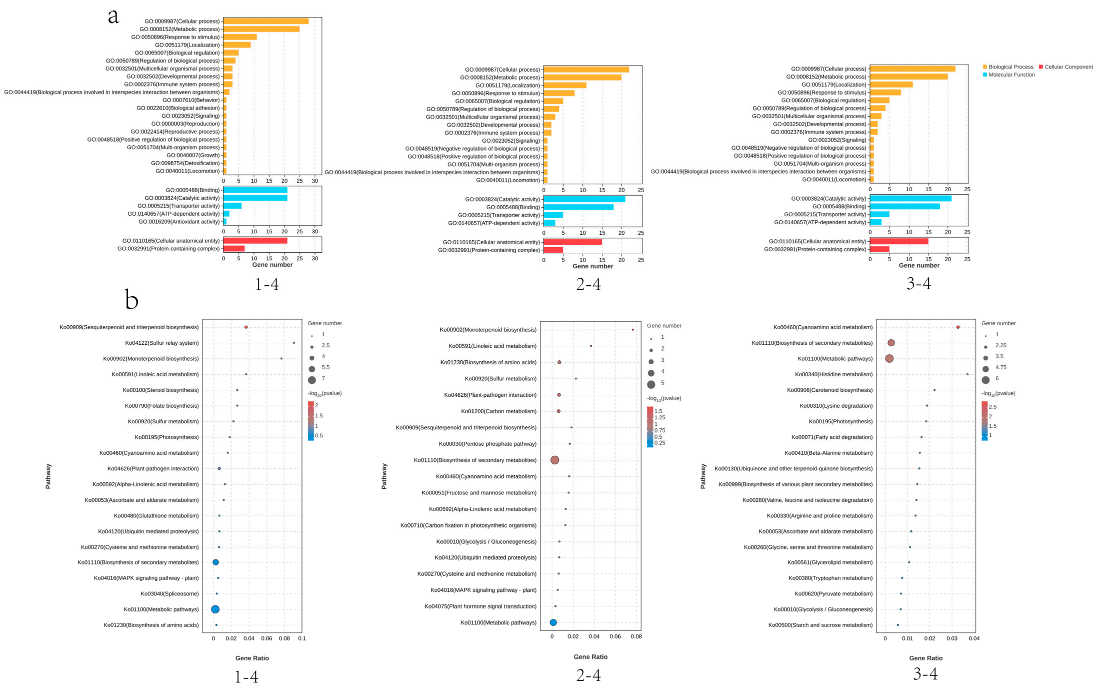

Supplementary Figure S1. (a) Bar chart for Gene Ontology enrichment analysis of three comparison groups. (b) Significance bubble plots for Kyoto Encyclopedia of Genes and Genomes (KEGG) analysis of three comparison groups.

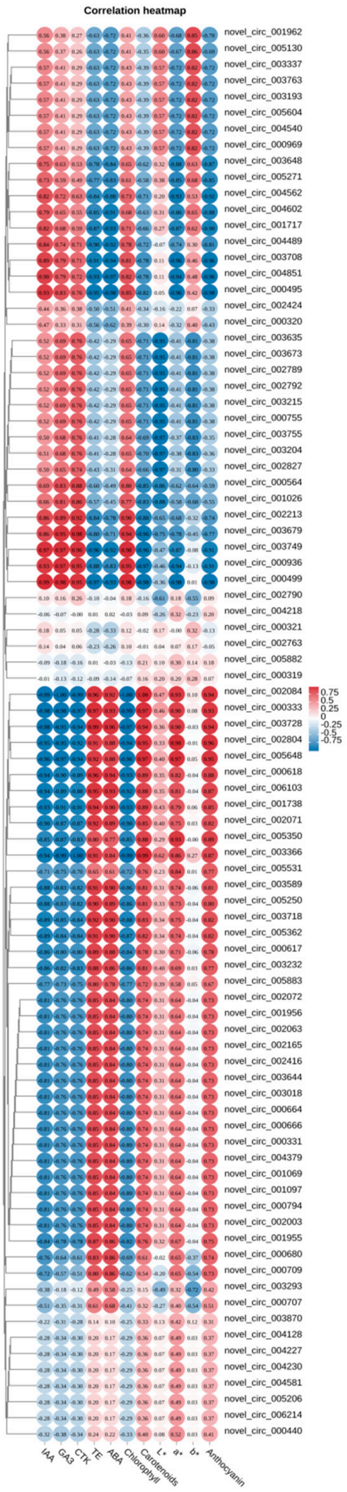

Supplementary Figure S2. Heat map of leaf color parameters, pigment content and five phytohormones in relation to circRNAs.
